# Assessment of infection in newly diagnosed multiple myeloma patients: risk factors and main characteristics

**DOI:** 10.1186/s12879-020-05412-w

**Published:** 2020-09-23

**Authors:** Chenyao Lin, Hui Shen, Shuimei Zhou, Minghui Liu, Anjie Xu, Shuang Huang, Changxin Shen, Fuling Zhou

**Affiliations:** 1 Department of Clinical Laboratory, Ningbo Medical Treatment Center Lihuili Hospital, Ningbo, P.R. China; 2 Genetic Diagnosis Centre, Zhongnan Hospital of Wuhan University, Wuhan, Hubei P.R. China; 3 Department of Hematology, Zhongnan Hospital of Wuhan University, 169 Donghu Road, Wuhan, Hubei 430071 P.R. China; 4 Blood Transfusion Department, Zhongnan Hospital of Wuhan University, 169 Donghu Road, Wuhan, Hubei 430071 P.R. China

**Keywords:** Infection, Multiple myeloma, New diagnosis, Risk factors, Prognosis

## Abstract

**Background:**

Infection is a leading cause of morbidity and death in patients with multiple myeloma (MM). The increased susceptibility to infection is complicated and multifactorial. However, no studies have explored the spectrum and risk factors of infections in newly diagnosed MM patients at the first admission. This cross-sectional study aimed to provide ideas for the assessment, prevention and treatment of infection in newly diagnosed MM patients when admitted for the first time.

**Methods:**

Retrospectively, the data from electronic medical records for 161 patients newly diagnosed with MM from May 2013 to December 2018 were analysed. All the information was collected at the time of admission, and the patients had received no antineoplastic therapy previously. Independent risk factors of infection in multiple myeloma were determined by univariate and multivariate analysis.

**Results:**

Newly diagnosed patients with MM were highly susceptible to viruses (43.9%), especially Epstein-Barr virus (EBV) (24.4%) and hepatitis B virus (HBV) (17.1%). Advanced stage (ISS stage III, *P* = 0.040), more severe anaemia (Hb < 90 g/L, *P* = 0.044) and elevated CRP (> 10 mg/L, *P* = 0.006) were independent risk factors for infection. Moreover, infections represented a major survival threat to patients with newly diagnosed MM (*P =* 0.033), and the existence of risk factors for infection was significantly correlated with poor prognosis (*P =* 0.011), especially ISS stage III (*P* = 0.008) and lower haemoglobin level (*P* = 0.039).

**Conclusions:**

Newly diagnosed MM patients are highly susceptible to viruses. Advanced ISS stage, more severe anaemia and the elevation of CRP are independent risk factors of infection, which also have a strong impact on prognosis. Our results suggest that viral infection should be taken into account if antibacterial drugs are not effective, and the prevention of infection and improvement of prognosis should be paid more attention in newly diagnosed patents with advanced stage and more severe anaemia.

## Introduction

Multiple myeloma (MM) is a malignant proliferating disease of plasma cells characterized by bone pain, anaemia, renal insufficiency and hypercalcemia. Clonal plasma cells in bone marrow proliferate abnormally and secrete monoclonal immunoglobulin or M protein, resulting in damage to related organs or tissues. MM, as the second most common haematologic neoplasm, accounts for approximately 2% of cancer-related mortalities.

Infection is a significant cause of morbidity and a principal cause of death in patients with MM [1–5]. Augustson et al. [6] observed that almost 50% of early deaths (< 6 months) were associated with infections in a study of over 3000 newly diagnosed MM patients. The increased susceptibility to infection in MM patients is complicated and multifactorial, probably due to the disease-related deficits in the innate or adaptive immune system, including hypogammaglobulinaemia [7–9]; numerical and functional abnormalities of dendritic cells [10], T cells [11] and natural killer cells [12]; and renal function impairment [7]. Beyond the inherent immune deficiency, some surveys described a changing spectrum of infections in MM, perhaps related to the different stages of treatment [13–15] and the more innovative treatment approaches of recent years, such as proteasome inhibitors (PIs), immunomodulatory drugs (IMiDs) and autologous stem cell transplantation (ASCT) [14, 16, 17].

In a recent study [18], the risk of developing a bacterial infection increased 7-fold and viral infections 10-fold in MM patients compared with matched controls. Previous studies have also indicated that Epstein-Barr virus (EBV) infection was more likely with patients with MM and monoclonal gammopathy of undetermined significance (MGUS) [19, 20]. In addition, higher hepatitis B virus (HBV) infection rates have been found in MM patients [21, 22]. Historically, the infections were most prevalent in untreated patients or patients on early induction therapy [23–25]. Therefore, doing research into the infection of newly diagnosed patients with MM and preventing death from infections are paramount. Huang et al. [26] followed up the patients who had blood stream infection (BSI) within 90 days after the diagnosis of multiple myeloma, finding that advanced ISS stage (ISS stage III) and poor ECOG performance status (ECOG> 2) were the independent risk factors of BSI. Coagulase-negative staphylococcus and *Escherichia coli* were the main pathogens. However, in past research, infection spectrum and risk factors have not been discussed in newly diagnosed MM patients when admitted. For the first time, we used a cross-sectional research design to study newly diagnosed MM patients at the initial visit. None of the patients had received antineoplastic therapy previously, and all the information, including the findings on infections, was collected at admission.

In this study, we comprehensively analysed a variety of clinical and laboratory parameters associated with infectious complications to describe the characteristics and identify the risk factors of infection in newly diagnosed MM patients. At the same time, we explored the impact of risk factors on survival. The ultimate purpose is to help develop strategies for the assessment, prevention and treatment of infection at the time of first admission to improve the prognosis of newly diagnosed patients with MM.

## Methods

### Patients

We took retrospective data from electronic medical records for 161 patients newly diagnosed with multiple myeloma who were first hospitalized in our department from May 2013 to December 2018. None of the patients had received antineoplastic therapy previously. The diagnosis of MM was based on International Myeloma Working Group (IMWG) criteria [27]. This study was approved by the relevant ethics committees/institutional review boards.

For all included patients, we obtained information on demographics, such as age, sex and Eastern Cooperative Oncology Group (ECOG) score. The infection-related data included microbial species, infection sites, neutrophils, lymphocytes, C-reactive protein (CRP) and invasive operation. The indicators related to MM disease included RBC, haemoglobin, albumin, globulin, serum creatinine, serum calcium, β2-microglobulin (β2-MG), lactate dehydrogenase (LDH) and clinical features (mainly including immunophenotype, Durie-Salmon stage, International Staging System stage, bone destruction, complications, comorbidities and survival time). The above information, including the findings on infections, was gathered at the time of admission. Patients were followed until 31 December 2018 or death, whichever came first.

### Measurements and definitions

The criteria for infection used in our study were the existence of a pathogen and imaging evidence of infection combined with concomitant clinical symptoms, such as non-pharmacological rise in body temperature (>37 °C), cough with sputum, painful urination and so on. Bacteria and fungi were identified by morphological, biochemical and serological reactions after isolation, purification and cultivation. Viral infections were diagnosed using polymerase chain reaction (PCR) technology to amplify DNA or RNA, such as for EBV, HBV and hepatitis C virus (HCV). Meanwhile, indirect immunofluorescence assays and chemiluminescence microparticle immunoassays were used to detect the IgM antibodies against respiratory syncytial virus (RSV), adenovirus, influenza virus, cytomegalovirus (CMV) and so on.

The cases were grouped as microbiologically defined infections (MDIs) when the microbiological assay of blood or secretion samples from any site indicated pathogen infections. Simultaneously, the cases were grouped as clinically defined infections (CDIs), when the results of the microbiological assay were negative but imaging evidence and clinical symptoms of infection existed. The MDIs and CDIs constituted the group with infections, and the other cases without the proof of infection were classified as the group without infections.

### Statistical analyses

All analyses were performed using SPSS 21.0. Comparisons between groups for categorical variables were performed using the chi-square test with Yates’s correction or Fisher’s exact test, as appropriate. Univariate analysis of infection rates was also performed by the chi-square test to screen the influencing factors of infection. The factors with *P* < 0.05 were selected and included in the multivariate analysis, which was performed using a binary logistic regression model (forward LR). From the multivariate analysis, potential confounding factors and multicollinearity were evaluated, and factors closely associated with other significant factors were excluded. *P* < 0.05 was considered to be statistically significant. The survival curves were calculated using the Kaplan-Meier method and were compared using the log-rank test. A *P* value of < 0.05 was defined as statistically significant.

## Results

### Patients’ characteristics

Overall, we analysed 161 patients for the first hospitalization with newly diagnosed multiple myeloma (94 males and 67 females). The average age was 64 years (range of 41–85), and 67.3% of patients were ≥ 60 years. One hundred patients had an ECOG score of 0 or 1, while 61 patients had a score ≥ 2. A total of 70 patients (43.5%) had MM of the IgG type, 30.4% of IgA type and 19.9% of light-chain type. On the Durie-Salmon (DS) scale, 10 patients were stage I, 26 were stage II, and 122 were stage III. On the International Staging System (ISS) scale, 10 patients were stage I, 46 were stage II, and 101 were stage III.

Among all patients, 147 (91.3%) had anaemia, 99 (61.5%) had bone destruction, and 52 (32.3%) had renal dysfunction, as the main complications. Hypertension (63 cases, 39.1%) and diabetes (24 cases, 14.9%) were the main comorbidities. Infections were found in 126 of 161 (78.3%) newly admitted patients. Of these, 31 (24.6%) were microbiologically defined (MDI), and 95 (75.4%) were clinically defined (CDI). Thirty-five patients were uninfected.

### Distribution of the infection sites

**Fig. 1:**
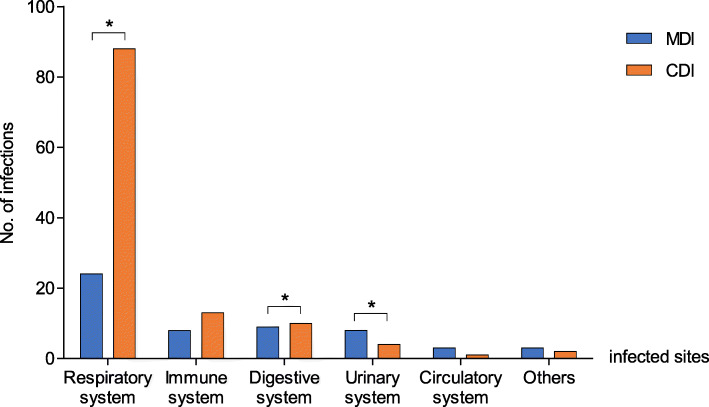
Distribution of infected sites in patients with multiple myeloma. There was a significant difference in the proportion of microbiologically defined infections (MDI) and in the proportion of clinically defined infections (CDI) in patients with respiratory, digestive or urinary system infections. *P < 0.05

A total of 173 infection sites were found in the 126 patients with infection. The most common site of infection was the respiratory system, in 112 cases (64.7%), followed by the immune system (21 cases, 12.1%), digestive system (19 cases, 11.0%), urinary system (12 cases, 6.9%) and circulatory system (4 cases, 2.3%). The others included 3 cases of alveolar osteitis and 2 cases of infection at an unknown site. In 43/126 cases (34.1%), more than 1 site of infection was found. CDI accounted for a higher proportion of respiratory infections (*P* = 0.044). MDI mainly appeared in the infection of urinary and digestive systems (*P* = 0.001, *P* = 0.027, respectively). The distribution of infected sites is summarized in Fig. 1.

### Distribution of the pathogens

Forty-one pathogens causing infections were identified in this sample. Unexpectedly, viruses (43.9%) were the main cause of infection. Bacteria (36.6%) followed, including 22.0% gram-negative bacteria and 14.6% gram-positive bacteria. Fungus accounted for 19.5%. Only 3 cases of blood culture were positive, for *Escherichia coli*, *Viridans streptococci* and *Ochrobactrum anthropic*, respectively.

It is worth mentioning that the constituent ratio of EBV (24.4%) was the highest out of all of the pathogenic microorganisms. HBV (17.1%) was in second place, and *Escherichia coli* (12.2%) and *Candida albicans* (12.2%) ranked third. Furthermore, 4 cases of herpes zoster infection were clinically diagnosed but without microbiological examination for herpes zoster virus (HZV). Therefore, it was not included in the list of pathogens. The constituent ratios of causative agents are shown in Table 1.

**Table 1:** Constituent ratios (%) of pathogens in patients with multiple myeloma

Pathogen	n	Constituent ratio (%)
Virus	**18**	**43.9%**
Epstein-Barr virus, EBV	10	24.4%
Hepatitis B virus, HBV	7	17.1%
Hepatitis C virus, HCV	1	2.4%
Fungus	**8**	**19.5%**
*Candida albicans*	5	12.2%
*Candida tropicalis*	1	2.4%
*Aspergillus*	1	2.4%
*Kodamaea ohmeri*	1	2.4%
Gram-negative bacteria	**9**	**22.0%**
*Escherichia coli*	5	12.2%
*Acinetobacter baumannii*	1	2.4%
*Pseudomonas aeruginosa*	1	2.4%
*Acinetobacter lwoffii*	1	2.4%
*Ochrobactrum anthropi*	1	2.4%
Gram-positive bacteria	**6**	**14.6%**
*Enterococcus faecalis*	2	4.9%
*Viridans streptococci*	2	4.9%
*Streptococcus pneumoniae*	1	2.4%
*Enterococcus faecium*	1	2.4%
Total	**41**	**100.0%**

The vast majority of patients with microbiologically defined infections were infected with only one pathogen (25/31 cases). Fewer than a quarter of microbiologically defined patients suffered more than one pathogen attack. Among them, there 3 cases of infections with two pathogens, 2 cases with three, and 1 case with four.

### Influencing factors of infection

According to the univariate analysis (Table 2), the factors associated with more frequent infections were poor performance status (ECOG> 2, *P* = 0.038), advanced stage (Durie-Salmon stage III, *P* = 0.011; ISS stage III, *P* = 0.005), more severe anaemia (Hb < 90 g/L, *P* = 0.014) and elevated CRP (> 10 mg/L, *P* = 0.007). Nevertheless, neutropenia (ANC < 1.5 × 10^9^ vs. ≥1.5 × 10^9^, *P* = 1.000) and lymphocytopenia (ALC < 1.0 × 10^9^ vs. ≥1.0 × 10^9^, *P* = 0.055) did not display significant differences.

**Table 2:** Univariate analysis of the infections in the MM patients

Factors	Investigated cases (*n* = 161)	Group with infections (%) (*n* = 126)	Group without infections (%) (*n* = 35)	χ2	*P* value
Sex				3.132	0.077
Male	94	69 (73.4)	25 (26.6)		
Female	67	57 (85.1)	10 (14.9)		
Age				2.583	0.108
<60	51	36 (70.6)	15 (29.4)		
≥ 60	110	90 (81.8)	20 (18.2)		
ECOG score				4.294	0.038
≤ 2	100	73 (73.0)	27 (27.0)		
>2	61	53 (86.9)	8 (13.1)		
Immunophenotype				0.672	0.880
IgG	70	54 (77.1)	16 (22.9)		
IgA	49	39 (79.6)	10 (20.4)		
Light chain	32	26 (81.3)	6 (18.8)		
Others	10	7 (70.0)	3 (30.0)		
Durie-Salmon stage				6.540	0.011
Stage I-II	36	23 (63.9)	13 (36.1)		
Stage III	122	102 (83.6)	20 (16.4)		
ISS stage				7.728	0.005
Stage I-II	56	37 (66.1)	19 (33.9)		
Stage III	101	86 (85.1)	15 (14.9)		
RBC (/L)				0.448	0.503
< 3.8 × 10^12^(f);	153	121 (79.1)	32 (20.9)		
< 4.3 × 10^12^(m) ≥3.8 × 10^12^(f); ≥4.3 × 10^12^(m)	8	5 (62.5)	3 (37.5)		
Haemoglobin (g/L)				6.092	0.014
<90	98	83 (84.7)	15 (15.3)		
≥ 90	63	43 (68.3)	20 (31.7)		
ANC (/L)				0.000	1.000
<1.5 × 10^9^	19	15 (78.9)	4 (21.1)		
≥ 1.5 × 10^9^	142	111 (78.2)	31 (21.8)		
ALC (/L)				3.676	0.055
< 1.0 × 10^9^	38	34 (89.5)	4 (10.5)		
≥ 1.0 × 10^9^	123	92 (74.8)	31 (25.2)		
CRP (mg/L)				7.235	0.007
≤ 10	95	69 (72.6)	26 (27.4)		
>10	62	56 (90.3)	6 (9.7)		
Albumin (g/L)				3.155	0.076
<35	111	91 (82.0)	20 (18.0)		
≥ 35	49	34 (69.3)	15 (30.6)		
Globulin (g/L)				0.056	0.813
≤ 30	39	31 (79.5)	8 (20.5)		
>30	121	94 (77.7)	27 (22.3)		
Serum creatinine (μmol/L)				0.450	0.502
<177	117	90 (76.9)	27 (23.1)		
≥ 177	44	36 (81.8)	8 (18.2)		
Serum calcium (mmol/L)				0.651	0.420
≤ 2.75	149	115 (77.2)	34 (22.8)		
>2.75	12	11 (91.7)	1 (8.3)		
β2-MG (mg/L)				1.805	0.179
<5.5	69	51 (73.9)	18 (26.1)		
≥ 5.5	87	72 (82.8)	15 (17.2)		
LDH (U/L)				0.124	0.725
≤ 243	105	85 (81.0)	20 (19.0)		
>243	32	25 (78.1)	7 (21.9)		
Bone destruction				3.581	0.058
Yes	99	82 (82.8)	17 (17.2)		
No	60	42 (70.0)	18 (30.0)		
Invasive operation				1.193	0.275
Yes	138	110 (79.7)	28 (20.3)		
No	23	16 (69.6)	7 (30.4)		
Renal dysfunction				1.213	0.271
Yes	52	38 (30.2)	14 (40.0)		
No	109	88 (69.8)	21 (60.0)		
Cardiac dysfunction				0.373	0.541
Yes	31	23 (18.3)	8 (22.9)		
No	130	103 (81.7)	27 (77.1)		
Hypertension				0.814	0.367
Yes	63	47 (37.3)	16 (45.7)		
No	98	79 (62.7)	19 (54.3)		
Diabetes				0.915	0.229
Yes	24	17 (13.5)	7 (20.0)		
No	137	109 (86.5)	28 (80.0)		

*ECOG* Eastern Cooperative Oncology Group, *ISS* International Staging System, *RBC* Red Blood Cells, *f* female, *m* male, *ANC* Absolute Neutrophil Count, *ALC* Absolute Lymphocyte Count, *CRP* C-reactive protein, *β2-MG* β2-microglobulin, *LDH* lactate dehydrogenase

Reference ranges of the laboratory values in our study: RBC 3.8 ~ 5.1 × 10^12^ (f), 4.3 ~ 5.8 × 10^12^/L(m); CRP ≤ 10 mg/L.

Multivariate logistic regression analysis (Table 3) demonstrated that advanced stage (ISS stage III, *P* = 0.040), more severe anaemia (Hb < 90 g/L, *P* = 0.044) and elevated CRP (> 10 mg/L, *P* = 0.006) were independent risk factors for infections. There was no collinearity among the factors (VIF < 2, tolerance > 0.85, respectively).

**Table 3:** Multivariate analysis of the infections in the MM patients

Factors	*P* value	OR	95% CI
ISS (I-II vs. III)	0.040	2.555	1.042–6.264
Hb (< 90 vs. ≥90 g/L)	0.044	2.554	1.027–6.350
CRP (≤10 vs. > 10 mg/L)	0.006	4.519	1.542–13.248

**Fig. 2:**
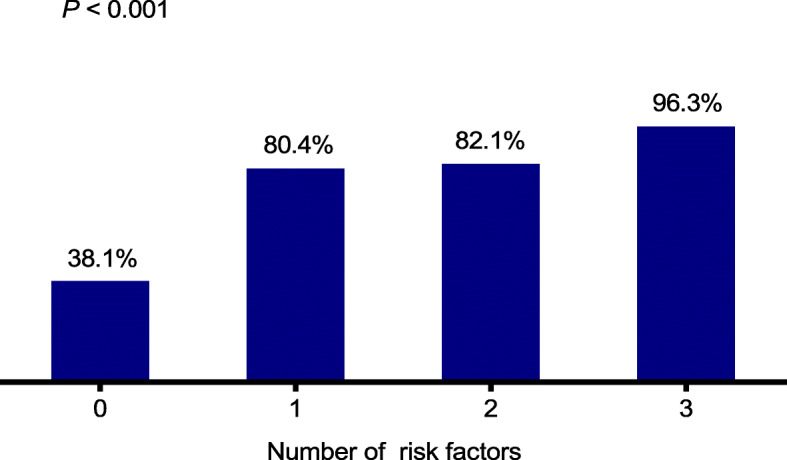
Infection rates corresponding to the number of independent risk factors. The three risk factors are advanced stage (ISS stage III), more severe anaemia (Hb < 90 g/L) and elevated CRP (> 10 mg/L)

The probability of infection was 38.1% in the absence of risk factors, 80.4% with 1 risk factor, 82.1% with 2 risk factors, and 96.3% with 3 risk factors (*P* < 0.001, Fig. 2). Visibly, the occurrence of independent risk factors significantly increased the infection rate (risk factors Yes vs. No, *P* < 0.001).

### Outcome analysis

**Fig. 3:**
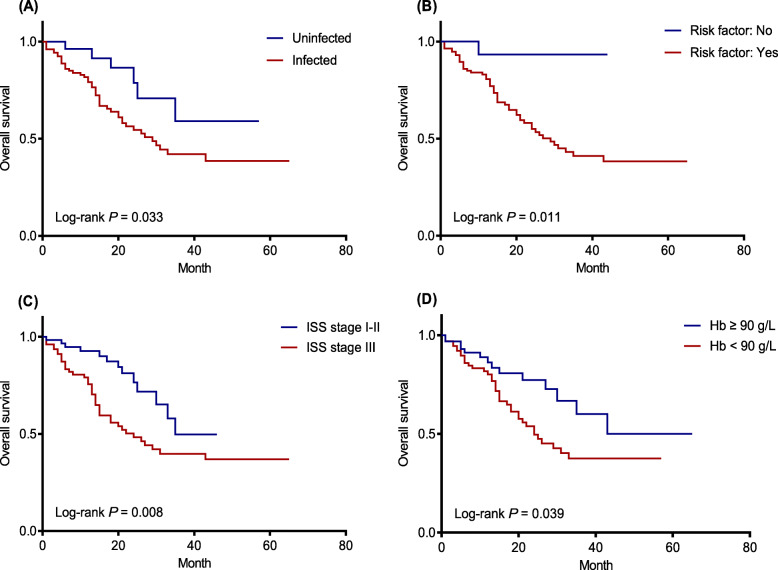
Assessment of overall survival in 161 newly diagnosed MM patients according to infections (**a**), risk factors (**b**), ISS stage (**c**) and serum haemoglobin (**d**). Yes: patients with any risk factor; No: patients with no risk factor. The risk factors are advanced stage (ISS stage III), more severe anaemia (Hb < 90 g/L) and the elevation of CRP (> 10 mg/L)

We counted and analysed the infections at the first hospitalization in newly diagnosed patients with MM to draw the Kaplan-Meier survival curve (Fig. 3a). The infected group showed a significantly shorter median OS compared with the uninfected group (29 months vs. not reached, *P =* 0.033). In our study, only one death occurred in the newly diagnosed patients without risk factors, and we found that the median OS of the patients with independent risk factors was significantly shorter than that without independent risk factors (29 months vs. not reached, *P =* 0.011) (Fig. 3b). Further analysis showed that among the risk factors, advanced stage (ISS stage III) and more severe anaemia (Hb < 90 g/L) were related to shorter survival time. Patients with ISS stage III had an obviously shorter median OS than those with ISS stage I-II (24 months vs. 35 months, *P =* 0.008) (Fig. 3c). In addition, the median OS of patients with low serum haemoglobin level (Hb < 90 g/L) was apparently shorter than that of patients with Hb ≥ 90 g/L (24 months vs. not reached, *P =* 0.039) (Fig. 3d).

## Discussion

Patients with MM suffer immune deficiency to varying degrees, which increases the risk of severe infections [3, 4, 28]. Similar to previous studies [6, 25, 26, 29–32], this study found that infection was common in newly diagnosed patients with MM and appeared to be the initial manifestation and the leading cause of poor prognosis. To provide ideas for the assessment, prevention and treatment of infection in newly diagnosed MM patients, we investigated the spectrum and risk factors of infection in 161 newly diagnosed MM patients at admission by a cross-sectional study.

According to our data, infections occurred in 78.3% of newly diagnosed patients with MM in our ward from May 2013 to December 2018. Respiratory infections were in the majority (64.7%), which was in concordance with the existing data [13, 26, 29, 30]. Urinary and digestive systems were more likely to cause microbiologically defined infections, mainly with *Escherichia coli*. Additionally, we found that viral (43.9%) and bacterial infections (36.6%) represented a major threat to MM patients, as also reported by Blimark et al. [18]. The main pathogenic bacterium in our study was also *Escherichia coli*, as in previous reports [13, 23, 26, 33]. *Candida albicans* was the main pathogenic fungus, consistent with past reports [34]; however, the infection rate by fungi (5.0%) was lower than a reported rate in patients receiving antitumour therapy (12.3%). Here, viruses were the most common pathogens, mainly including EBV and HBV, in contrast to published research [13, 33, 35, 36], which found that gram-negative bacteria were the leading pathogens and herpes zoster virus was the main pathogenic virus. Based on data on antitumour therapy in previous studies, chemotherapy can increase the risk of bacterial infection [13], and bortezomib can increase the risk of herpes zoster virus infection [16, 17, 37]; therefore, the difference in infection spectrum may be related to the therapeutic factors. In addition, the sample size of our study was relatively small, which might have led to bias in the results.

Patients with multiple myeloma are more susceptible to viral infection [38]. Blimark et al. [18] showed that the risk of developing viral infection in patients with MM was 10 times higher compared with matched controls. Through molecular analysis, a recent study [19] displayed significant EBV DNA in malignant plasma cell disorders, especially in MM and MGUS patients. Bosseboeuf et al. [20] demonstrated that EBV was the most frequent target of purified monoclonal IgG produced by patients with MGUS or MM. They considered that chronic stimulation by infectious Ag may promote MGUS and MM. It can be concluded that EBV is associated with MM. An early study [39] confirmed that HBV was lymphotropic and was able to infect and replicate in human lymphocytes and monocytes. A study in Japan [21] reported that the rate of HBV infection in patients with MM was 3.2%, higher than that in the group of healthy subjects (1.2%). Huang et al. [22] found that patients with MM had a significantly higher HBV carrier rate than patients with acute leukaemia and that patients with MM who were HBV carriers were at significantly higher risk of having hepatic injury than non-carriers. Our research was carried out in central China. The seroprevalence of EBV was similar to the global proportion [40, 41], and the prevalence of hepatitis B was intermediate (5.23, 95% CI: 3.11–7.34%) [42]. Coinciding with the views of early researchers, we believe that viruses play an important role in patients with MM, especially EBV and HBV in our study. Therefore, the prevention and treatment of the virus in newly diagnosed patients with MM is essential. If an underlying chronic infection is cleared up early enough in disease progression, it could offer the possibility of preventing MGUS transition to SM and MM in the first place [20]. In addition, we agree with the point that newly diagnosed patients with MM should be screened for serum hepatitis B viral markers universally in HBV endemic areas [22]. In China, HBV markers have been screened in newly diagnosed MM patients with a high likelihood of infection. Beyond that, we should pay more attention to strengthening the monitoring of HBV DNA.

In our research, according to the univariate and multivariate analysis, advanced ISS stage (ISS stage III), more severe anaemia (Hb < 90 g/L) and elevated CRP (> 10 mg/L) were identified as independent determinants of infection patients with MM. Meanwhile, poor performance status (ECOG> 2) and advanced DS stage (DS III) were the influencing factors of infection. ANC and ALC did not display significant differences. Huang et al. [26] recently showed that ISS stage III and ECOG> 2 were the independent risk factors of BSI in patients with newly diagnosed MM, and more severe anaemia (Hb < 100 g/L) and worse renal function (Cr ≥ 177 μmol/L) were influencing factors associated with BSI. ALC showed no significant difference. However, they did not include DS stage, ANC or CRP in their univariate or multivariate analysis; the blood stream was the only discussed infection site; and patients received antitumour therapy, all of which may have caused the difference in risk factors in the final model between our studies.

ISS is a widely accepted staging system based on serum levels of albumin and β2-MG [43]. Serum albumin level is inversely correlated with healthy diet and has been recognized as a sign of rapid tumour growth [44]. In addition, serum β2-MG level is elevated in patients with MM due to renal insufficiency as well as tumour burden. In an unselected cohort, Caravita et al. [45] reported that only ISS stage was a risk factor affecting severe infection development. Isoda et al. [46] indicated that advanced ISS stage was an independent risk factor associated with severe (grade C 3) bacterial infection in MM patients. A large number of studies [13, 26, 33] have shown that advanced ISS stage is an important risk factor for infection in MM patients. In agreement with previous reports, advanced ISS stage appeared to be associated with a higher incidence of infection in our cases. We hold the opinion that patients with ISS stage III, mostly morbid patients with high disease activity, have poorer prognoses and are susceptible to serious infection complications [46].

The decrease in haemoglobin content, on the one hand, reduces the concentration of respiratory enzymes, mitochondrial oxidase and myoglobin, resulting in a deficient oxygen supply, decreased aerobic metabolism and accumulation of lactic acid. On the other hand, it affects the immune response and phagocytosis, which in turn leads to the depression of immune functions and disturbances of immune regulation, subsequently increasing the risk of infection [47]. It has been found that anaemia is a risk factor for accompanying infection in patients with MM. Dumontet et al. [48] included haemoglobin in the predictive model of first treatment-emergent (TE) grade ≥ 3 infection in the first 4 months in patients with MM. TE infections were defined as infections that occurred or worsened on or after the first dose of any drug and within 28 days after discontinuation of treatment. Similar to early research, lower haemoglobin levels also showed a significant correlation with infection in our study. Therefore, according to the European Myeloma Network [49], we consider that patients with persistent symptomatic anaemia (haemoglobin < 10 g/dL) without other causes may initiate treatment with erythropoietic-stimulating agents.

CRP is an acute-phase reaction protein (APRP) synthesized by liver cells in response to inflammatory stimuli such as microbial invasion or tissue damage. CRP increases within the first few hours of inflammation and peaks at 48 h, which is not affected by radiotherapy, chemotherapy or corticosteroid therapy. Rintala et al. [50] demonstrated that CRP was a reliable and readily available method to differentiate between bacterial infections and other causes of fever in patients with malignant haematological diseases. Apewokin et al. [51] concluded that the elevated CRP in patients with haematological malignancies could be used as a sensitive screening index for viral infection. In our research, the elevation of CRP was correlated with the risk of infections, and 12 (57.1%) patients with viral infection were accompanied by elevated CRP, while the rate of increased CRP was 38.6% in patients without viral infection. Therefore, for severely infected patients with elevated CRP, if using antibacterial drugs is not effective, the viral infection should be taken into account.

Numerous previous studies have suggested that neutropenia is a risk factor for infection with MM. High-dose alkylating agents and new drugs can lead to myelosuppression and agranulocytosis, especially in combination [52]. In this study, absolute neutrophil count (ANC) data before treatment showed that 80.1% (100/126) of infected patients had a normal ANC, and neutropenia was present in only 11.9% (15/126). Although the occurrence of neutropenia may also be due to the disease itself, that was not reflected in this study, possibly because the remaining myeloid progenitor cells of MM patients still balanced the production and consumption of neutrophils, or the stored mature neutrophils in bone marrow still replenished the cells in the circulation. Moreover, haematopoietic function was suppressed in patients with MM, and neutrophilia was not necessarily observed during infection. Only 8.7% (11/126) of the patients had neutrophilia in our analysis. As a result, for newly diagnosed MM patients, the decrease or increase in ANC cannot be used as an indicator of infection. The absolute lymphocyte count (ALC), as a marker of host immunity, has been widely studied in a variety of malignancies. Although its role in infections in newly diagnosed MM patients remains indeterminate, the significance on infection risk and survival has been described in MM patients during bortezomib treatment [35, 53]. However, ALC did not show any marked difference in infections in this research, which may be attributable to the fact that patients had not undergone previous treatment.

Newly diagnosed patients with MM have variable survival, ranging from a few days to more than a decade [54, 55]. Many studies [1, 2, 6, 18, 31, 35] confirm that infections represent a major threat to patients with MM. Caravita et al. [45] attested that the overall survival (OS) of MM patients with infections was significantly shorter than in those without infections. The same conclusion was reached in our study of newly diagnosed MM patients with infection at admission compared with those without infection (*P* = 0.033). The median OS of patients with independent risk factors was significantly shorter than in those without independent risk factors (*P* = 0.011). Among the risk factors, mainly advanced stage (ISS stage III, *P* = 0.008) and more severe anaemia (Hb < 90 g/L, *P* = 0.039) were significantly associated with poor prognosis. It can be seen that infection at admission is a significant cause of poor prognosis in newly diagnosed patients with MM, and the existence of independent risk factors of infection seriously affects the prognosis of newly diagnosed MM patients, especially ISS stage III and lower haemoglobin level.

There are several limitations to our research. First, the retrospective design may have led to biased selection of patients, and the number of participants was small. In addition, the performance of interphase fluorescence in situ hybridization (iFISH) in MM patients is not common for various reasons, such as the expensive price, resulting in a serious lack of information on cytogenetics, so the data of the r-ISS stage were not analysed in our study. However, considering the broadscale clinical application, ISS stage may be of a higher practical value in predicting infection at present. Because of its important role in evaluating the prognosis of patients with MM, R-ISS also needs to be included in analyses along with the popularization of cytogenetic detection technology.

## Conclusions

Newly diagnosed patients with MM are highly susceptible to viruses, especially Epstein-Barr virus and hepatitis B virus. Advanced ISS stage (ISS stage III), more severe anaemia (Hb < 90 g/L) and elevated CRP (> 10 mg/L) were identified as independent risk factors for infection. Infections represented a major threat to patients with newly diagnosed MM, and the existence of risk factors of infection had a strong impact on the prognosis, especially ISS stage III and lower haemoglobin level.

## Data Availability

The datasets used and/or analysed during the current study are available from the corresponding author on reasonable request.

## Abbreviations

MM: Multiple Myeloma
BSI: Blood Stream Infections
EBV: Epstein-Barr virus
HBV: Hepatitis B virus
HCV: Hepatitis C virus
RSV: Respiratory Syncytial virus
CMV: Cytomegalovirus
MDI: Microbiologically defined infections
CDI: Clinically defined infection
DS: Durie-Salmon
ISS: International Staging System
ECOG: Eastern Cooperative Oncology Group
RBC: Red Blood Cell
ANC: Absolute Neutrophil Count
ALC: Absolute Lymphocyte Count
CRP: C-reactive protein
β2-MG: β2-microglobulin
LDH: Lactate Dehydrogenase
OS: Overall Survival
MGUS: Monoclonal Gammopathy of Undetermined Significance
